# Differential electroencephalography responses in speech perception between native and non-native speakers

**DOI:** 10.3389/fnhum.2025.1661010

**Published:** 2025-10-24

**Authors:** Luong Do Anh Quan, Le Thi Trang, Inyong Choi, Jihwan Woo

**Affiliations:** ^1^Department of Electrical, Electronic and Computer Engineering, University of Ulsan, Ulsan, Republic of Korea; ^2^Department of Communication Sciences and Disorders, University of Iowa, Iowa City, IA, United States; ^3^Department of Biomedical Engineering, University of Ulsan, Ulsan, Republic of Korea

**Keywords:** temporal response function, phoneme-related potential, passive listening, neural tracking, bottom-up acoustic features

## Abstract

**Introduction:**

Native and non-native listeners rely on different neural strategies when processing speech in their respective native and non-native languages, encoding distinct features of speech from acoustic to linguistic content in different ways. This study investigated differences in neural responses between native English and Korean speaker when they passively listened to speech in their native and non-native languages using electroencephalography.

**Methods:**

The study employed two approaches to examine neural responses: Temporal Response Functions (TRFs) measure how the brain tracks continuous speech features (i.e., speech envelope, phoneme onset, phonemic surprisal, and semantic dissimilarity), and Phoneme-Related Potentials (PRPs) assess phonemic-level processes.

**Results:**

Non-native speakers showed significantly stronger neural tracking of the speech envelope, but no group differences for higher-level linguistic features within analyses of TRFs. PRP analyses, however, revealed distinct response patterns across phoneme categories, with non-native speakers showing heightened peaks.

**Conclusion:**

The results suggest that non-native speakers rely more on bottom-up acoustic cues during passive listening. TRFs and PRPs provide information on neural markers that indicate how speech is processed differently depending on the listener's native language and language experience.

## 1 Introduction

Speech perception is an important area of study within auditory neuroscience, particularly in exploring how differently listeners process their native vs. non-native languages and in understanding how language experience shapes neural responses. Previous studies using Electroencephalography (EEG) have investigated the mechanisms underlying the perception of different languages at the phonemic level, focusing on elements such as phonemes and consonant-vowel combinations (Babcock et al., 2012; Brandmeyer et al., 2013; Winkler et al., 1999). For instance, mismatch negativity, observed by contrasting Event-Related Potentials (ERPs) to the vowels, has been used to identify cognitive differences between native and non-native speakers (Winkler et al., 1999). Additionally, (Brandmeyer et al. 2013) employed decoding of consonant/vowel-evoked EEG signals to investigate differences in language comprehension between these groups.

Beyond phoneme-level processing, other studies have explored differences in sentence-level comprehension between native and non-native speakers, focusing on case markers—a linguistic tool indicating the grammatical role of nouns or pronouns—and listening effort (Babcock et al., 2012; Coughlin et al., 2019; Song and Iverson, 2018). Case marker violations have been used to elicit N400-P600 patterns during the processing of nominatives and accusatives (Mueller et al., 2007). Additionally, increased listening effort modulates auditory and lexical processing, as reflected in N400 amplitude (Song and Iverson, 2018). Although native and non-native speakers often exhibit similar ERP components, the responses in native speakers typically occur with longer latencies (Babcock et al., 2012; Coughlin et al., 2019). Prior studies often used short speech token stimuli, such as phonemes or consonant-vowel pairs, to efficiently measure ensemble-averaged ERPs (Martin et al., 2008), but this approach does not adequately capture neural activity in response to natural, continuous speech. To investigate the neural mechanisms of continuous-speech perception, it is essential to extract interpretable and meaningful EEG responses from continuous speech and identify the key components involved. (Marmarelis 2004) proposed that the human brain behaves as a linear time-invariant system and utilized reverse correlation to identify its Temporal Response Functions (TRFs) (Abrams et al., 2008; Ahissar et al., 2001; Ringach and Shapley, 2004; Marmarelis, 2004). This concept facilitated encoding of neural entrainments to speech stimuli using a speech-EEG synchrony approach referred to as neural tracking (Obleser and Kayser, 2019). Both slow temporal variations reflected in speech envelope (Aiken and Picton, 2008; Di Liberto et al., 2015; Muralimanohar et al., 2017; O'Sullivan et al., 2015) and rapidly changing temporal fine structure offer key cues necessary for accurate speech perception (Etard et al., 2022). An increase in neural tracking of the speech envelope has been particularly observed in non-native speakers (Reetzke et al., 2021; Song and Iverson, 2018). Neural tracking of other speech features, such as phoneme onsets, phonemic surprisal, and semantic dissimilarity, was also examined by (Di Liberto et al. 2021). They reported that the neural representations of these linguistic features in non-native speakers with more language proficiency become more similar to those of native speakers.

Previous literature has debated whether neural entrainment to the speech envelope is primarily driven by bottom-up processes or shaped by top-down predictive mechanisms, given that the envelope closely reflects the acoustic content of speech (Ding and Simon, 2014; Mesgarani and Chang, 2012; Millman et al., 2012). This debate extends to non-native language processing, particularly regarding how bottom-up and top-down mechanisms contribute to differences in envelope tracking between native and non-native speakers. Prior studies have reported differences in neural tracking of the speech envelope between native and non-native speakers in competing-speech paradigms (Song and Iverson, 2018; Zou et al., 2019). Such differences, however, may partly reflect downstream processes related to suppressing meaningful information in competing speech driven by top-down mechanisms, given that they used speech stimuli from the same language, rather than a direct effect of language experience. Enhanced neural tracking of the speech envelope has also been observed in an auditory selective attention paradigm where informational masking was minimized (Reetzke et al., 2021). These findings support an integrative incomplete language model, suggesting that non-native speakers, due to the underdeveloped internal language system, rely more strongly on bottom-up acoustic cues during listening, even though this may be insufficient for effective comprehension (Mattys et al., 2010, 2013).

Nonetheless, auditory selective attention paradigms may introduce confounds related to participants' ability to segregate concurrently incoming auditory streams. These confounds can be mitigated by employing an experimental paradigm with visual distractors instead of additional speech (Vanthornhout et al., 2019). To directly investigate how language experience influences neural entrainment to bottom-up acoustic features, this study employed a passive listening paradigm in which participants watched a silent movie while auditory stimuli were presented. This method minimizes top-down predictive influences, allowing for an implicit examination of the neural processes underlying speech perception (Vanthornhout et al., 2019). Passive listening paradigms better simulate real-world listening conditions and highlights the unconscious neural processes that differ between native-non-native speakers (Borghini and Hazan, 2018; Sun et al., 2023). Understanding these differences is crucial for developing effective approaches to enhance communication and to improve educational effects of learning a second-language (Fuhrmeister et al., 2023; Ihara et al., 2021).

Beyond envelope tracking, low-frequency neural activity has been shown to concurrently track linguistic information in speech as well (Keitel et al., 2018; Ding et al., 2016; Di Liberto et al., 2015). In particular, neural tracking to linguistic features such as phoneme onsets, phonemic surprisal, and semantic dissimilarity has been found to be modulated by language proficiency (Di Liberto et al., 2021). Accordingly, the present study aims to examine neural tracking to both the acoustic feature (i.e., speech envelope) and higher-level linguistic features (i.e., phoneme onsets, phonemic surprisal, and semantic dissimilarity) of speech in native and non-native speakers using a TRF approach. Given that passive listening predominantly reflects bottom-up processing, the neural responses observed are assumed to exhibit minimal dependence on language proficiency. We anticipate finding no differences in TRFs to linguistic features between the two groups, consistent with predominantly bottom-up processing during passive listening.

(Ge et al. 2015) demonstrated that distinct brain networks underlie speech comprehension across different language groups. This implies that neural processing differences between native and non-native speakers are not universal but rather depend on the characteristics of the specific language. Therefore, it is essential to expand research on how language-specific characteristics influence neural tracking in native and non-native speakers.

Given that TRF analysis is a multiple lagged regression-based method, the estimated TRF models depend on linear assumptions as well as the choice of regularization method and parameters. Complementary to TRF analysis, the temporal dynamics of neural responses to phonemes have been explored using a time-locked averaging approach, Phoneme-Related Potentials (PRPs), by marking each phoneme onset as an event (Khalighinejad et al., 2017). This method, the PRP analysis, therefore preserves the precise temporal properties of neural responses to phonemes (Khalighinejad et al., 2017). PRP components, appearing roughly 50–400 ms after phoneme onset, provide how specific phonetic information is encoded during speech comprehension. PRPs have recently been used to investigate the impact of speech degradation on phonemic encoding in individuals with normal hearing (Jeon and Woo, 2023), and to distinguish attending phonemic encoding in both normal-hearing and cochlear implant users (Aldag and Nogueira, 2024). Those studies revealed distinct PRP patterns across phonemic categories in different languages, indicating that PRP characteristics may be influenced by language-specific factors.

Building on the literature review, this study explores how native vs. non-native language experience influences neural processing during speech comprehension using two frameworks: PRPs and TRFs. These approaches offer a multidimensional perspective on how linguistic background affects both low-level and phonemic-level speech perception mechanisms. By incorporating both English and Korean sentence stimuli, this study aimed to cross-validate the influence of language type on neural processing under passive listening. It was hypothesized that non-native speakers with lack of language experience would show enhanced neural tracking of the speech envelope, while higher-level linguistic related processing would remain unaffected (Reetzke et al., 2021). Regarding PRPs, amplitude differences between native and non-native speakers were also anticipated. Moreover, differences in envelope tracking and phonemic level process were expected between non-native speakers of Korean and English, in accordance to prior evidence showing distinct neural networks involved in prosodic processing across languages (Ge et al., 2015).

## 2 Materials and methods

### 2.1 Experimental setup and EEG data collection

Ten English sentences from the Revised Speech Perception in Noise Test (R-SPIN; mean ± standard deviation [SD] of duration, 1.8 s ± 0.2) and ten Korean sentences from the Korean Standard Sentence List (mean ± SD of duration, 1.9 s ± 0.2) were used as speech stimuli (Bilger et al., 1984; Jang, 2008). Each sentence was randomly presented 50 times at a sound pressure level of 65 dBA via a loudspeaker, positioned 1 m apart from the participant. The inter-stimulus interval was set to 3 s. The experiment was conducted in a soundproof booth under passive conditions, which required participants to sit in a comfortable chair and watch a silent movie during the experiment.

Twenty English native speakers (mean ± SD of age, 23.5 ± 5.7; 3 males and 17 females) and twenty Korean native speakers (mean ± SD of age, 21.8 ± 1.8; 10 males and 10 females) participated in the experiment. The number of participants was decided to be comparable with previous related works (Reetzke et al., 2021; Song and Iverson, 2018; Zou et al., 2019). All participants reported limited experience in their respective non-native language. None of the English native participants lived in Korea for more than 6 months, and none of the Korean native participants lived in English-speaking countries for more than 6 months. No direct assessment of language proficiency was conducted. None of the participants had neurological conditions or disorders, and all the participants had normal or corrected-to-normal visual ability. The participants provided written informed consent prior to the experiment.

Data collection was conducted at two different sites under identical experimental settings: data from English native speakers were collected at the University of Iowa (Iowa City, IA, USA), and data from Korean native speakers were collected at the University of Ulsan (Ulsan, Republic of Korea). All study procedures were reviewed and approved by the Institutional Review Boards of the University of Iowa (201910839) and the University of Ulsan (1040968-A-2020-015), and the experimental procedures were performed in accordance with relevant guidelines and regulations.

### 2.2 EEG acquisition and pre-processing

Neural response to speech sentences was recorded using a 64-channel EEG Active2 system at a sampling rate of 2,048 Hz (Biosemi Co., Amsterdam, The Netherlands). The EEG signals were filtered using a zero-phase 1-Hz high-pass filter and a zero-phase 57-Hz low-pass filter. The high-pass filter had a narrow 0.5-Hz transition band to retain low-frequency neural activity, while the low-pass filter featured a wider 2-Hz transition band. The EEG data were down sampled to 256 Hz for computational efficiency and epoched with a duration of 3,000 ms, 500 ms prior to, and 2,500 ms after stimulus onset. Bad channels and bad epochs were rejected, and then the rejected channels were interpolated back into the EEG data. Eye activity and other artifacts were determined and removed using independent component analysis. Out-of-bound epochs with amplitudes exceeding 200 μV were removed. The EEG signals were finally filtered using a zero-phase 1–15-Hz band-pass filter (order 5), as low-frequency entrainment to speech has been shown to reflect both acoustic and linguistic feature processing (Di Liberto et al., 2015, 2021), and downsampled to 128 Hz. Pre-processing was performed using EEGLAB and custom MATLAB code (Delorme and Makeig, 2004).

### 2.3 Computation of speech features

Two categories of stimulus features were employed: (i) low-level acoustic features, represented by the speech envelope (Env); and (ii) higher-level linguistic features, including phoneme onset (Pon), phonemic surprisal (Ps), and semantic dissimilarity (Sem). The analytic speech envelope was derived using the Hilbert transform and subsequently filtered with a zero-phase 15-Hz low-pass filter (order 5). The filtered envelopes were down sampled to a frequency of 128 Hz to match the sampling rate of EEG data. Phoneme onsets from the sentences were extracted using Praat software (Boersma, 2001; Kisler et al., 2017; Yoon and Kang, 2014). Phonemic surprisal represents the degree of uncertainty of each phoneme, calculated as the probability of the current phoneme given the preceding phonemes for each word. To estimate the value of phonemic surprisal, the probability of the given word form was extracted based on the SUBTLEX database for Korean (Tang and de Chene, 2014) and English (Brysbaert and New, 2009). Semantic dissimilarity was used to measure the co-occurrence of each word with all other words within the sentence (Broderick et al., 2018). Here, the co-occurrence is captured based on Pearson's correlation between a feature vector for the given word and the mean feature vector averaged across all previous words in the sentence. These feature vectors, representing each word as a vector in a 400-dimensional space, were computed using a pre-trained word2vec model (Grave et al., 2018).

### 2.4 Computation of TRFs

**Figure 1:**
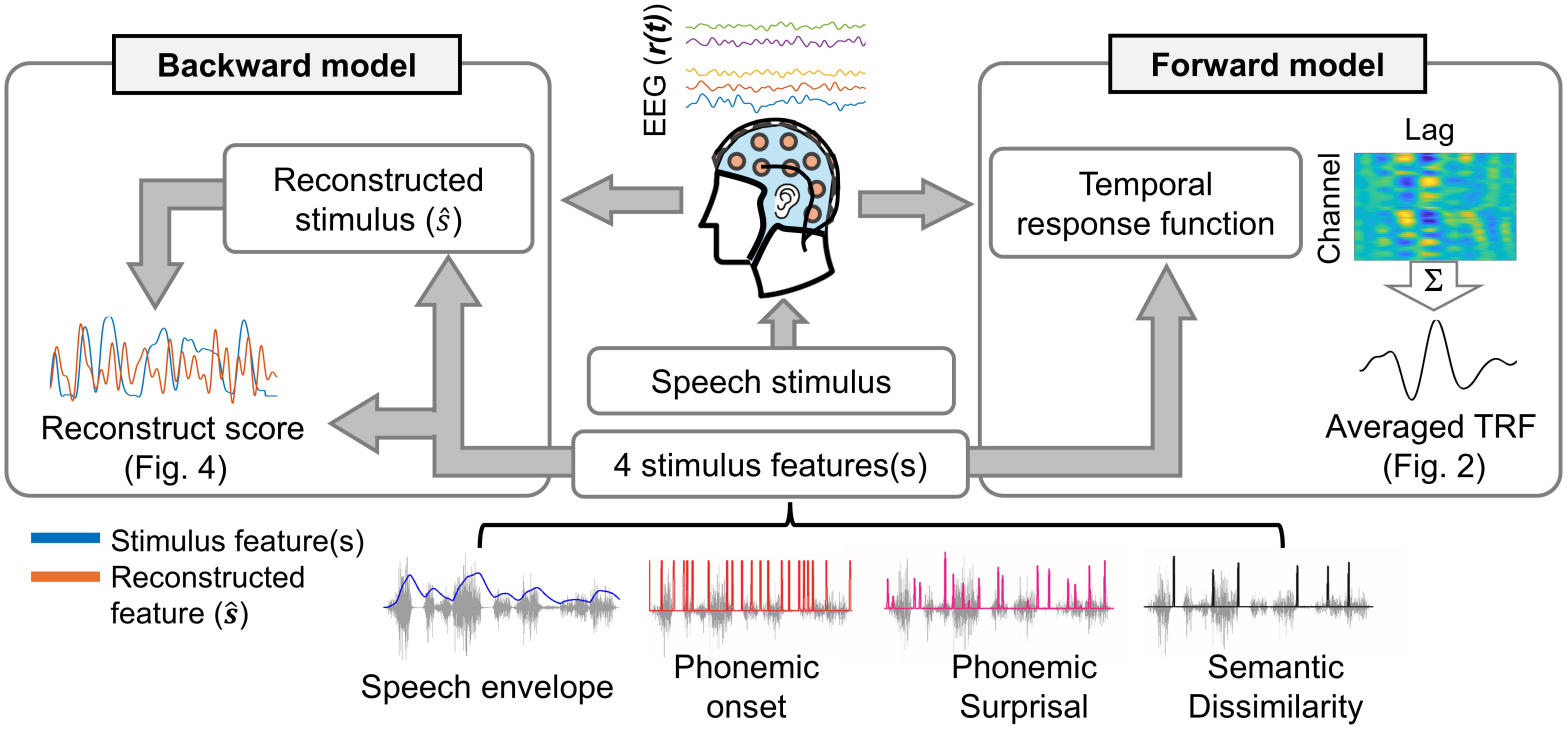
Scheme of TRFs fitting in both forward (left side) and backward (right side) models.

Figure 1 shows the procedure of estimating forward and backward models for the speech envelope, phoneme onset, phonemic surprisal, and semantic dissimilarity.

In this study, the TRF approach was employed to investigate neural entrainment to natural speech, as previous research has demonstrated its reliability and efficacy even in scenarios involving short sentences (Das et al., 2020; Muncke et al., 2022; Slaats et al., 2023; Vanthornhout et al., 2019). For each subject, forward and backward models were estimated by fitting TRFs independently to individual trials, after which the TRFs were averaged across trials.

The forward models use speech features as inputs to predict neural responses to speech stimuli by estimating the TRFs. The fit of speech features to EEG was computed using regularized linear regression (Crosse et al., 2016). The reliability of estimated forward models was evaluated by quantifying the EEG prediction correlation (Pearson's r). A leave-one-out cross-validation (across trials) procedure was applied to identify the optimal regularization parameters from a range of 10^−1^ to 10^6^ (Browne, 2000). The value of the regularization parameter that yielded the highest cross-validation performance was determined as the optimal parameter. These optimal regularization parameters were chosen separately for each speech feature model (i.e., envelope-based model, phoneme onset-based model, phonemic surprisal-based model, and semantic dissimilarity-based model). A time-lag window of −100 to 500 ms was used to fit all the TRF models.

The backward models, which use EEG as the input to synthesize speech features, were also computed using regularized linear regression (Crosse et al., 2016). The reliability of estimated backward models was evaluated by quantifying the stimulus reconstruction correlation (Pearson's r). Determination of the optimal regularization parameter followed the same procedure as that of the forward model. The efficiency of backward models was assessed using the reconstruction score, computed as the correlation between the reconstructed speech features and the corresponding ground truth. In fitting the target backward model, a time-lag window ranging from 0 to 500 ms was employed. Furthermore, baseline backward models, fitted using a time-lag window spanning from −100 to 0 ms, were used to evaluate the robustness and reliability of the target backward models in reconstructing various speech features.

### 2.5 Computation of PRPs

The utterance rates of all phonemes for Korean and English are summarized in Table 1. In this study, each sentence was played 50 times, giving the minimum utterance rate for each phoneme of 50. The EEG of each channel was normalized to have zero mean and unit variance. The neural response to each phoneme was computed by segmenting EEG signals relative to before/after the phoneme onset time to have an interval of −50 to 500 ms. The PRPs for each phoneme were then computed by averaging all neural responses to that phoneme. The grand average PRPs was computed by averaging all PRPs across all phonemes, all epochs, and all electrodes. The neural responses to groups of phonemes that share the same manner of articulation (vowel, nasal, plosive, and fricative) were also computed by averaging all PRPs across all phonemes in the corresponding group, all epochs, and all electrodes.

**Table 1:** Number of utterances for each phoneme in Korean and English sentences.

**Phoneme(number of utterances)**							
**Korean sentences**				**English sentences**			
**Vowel**	**Nasal**	**Plosive**	**Fricative**	**Vowel**	**Nasal**	**Plosive**	**Fricative**
YEO (150)	N (1150)	B (200)	SS (100)	AA (300)	M (400)	Y (50)	DH (400)
WO (100)	NG (300)	P (450)	S (500)	AO (100)	L (400)	W (200)	CH (150)
A (1000)	M (300)	K (500)	H (200)	AH (700)	N (550)	B (300)	Z (200)
E (400)	L (450)	KK (300)	J (550)	AW (150)	NG (200)	P (300)	S (600)
I (750)		D (100)	CH (150)	OW (150)	R (200)	G (150)	HH (100)
EO (500)		T (50)		AR (500)		T (850)	F (50)
YO (200)				EY (200)		D (350)	SH (100)
U (450)				AY (300)		K (500)	
EU (750)				EH (150)		V (50)	
AE (150)				UW (100)			
O (100)				IY (150)			
HA (100)				IH (500)			
WA (50)				ER (350)			
				UH (150)			
4,700	2,200	1,600	1,500	3,800	1,750	2,750	1,600
Total: 10,000				Total: 9,900			

The values in the parentheses represent the number of utterances.

### 2.6 Statistical analysis

The statistics employed in the current study include *t*-tests for assessing differences in TRF weights derived from forward models to various speech features and PRPs between native and non-native speakers in each language, as well as permutation tests for evaluating the robustness of target backward models compared to their corresponding baseline models and between the target backward models of native and non-native speakers.

Specifically, two-sample *t*-tests were used to evaluate differences in TRF weights for each speech feature across all channels and time points, as well as differences in PRPs, averaged over all channels and time points, between native and non-native speakers. The *p*-values from these *t*-tests were corrected for multiple comparisons using the False Discovery Rate (FDR) method: across all channels and time points for TRFs, and across all time points for PRPs (Benjamini and Hochberg, 1995; Benjamini and Yekutieli, 2001). For significant differences, *t*-values with 38 degrees of freedom were reported in absolute form (|t(38)|), alongside corrected *p*-values, since the effects were observed across multiple electrodes and/or time points. Dominance was determined by comparing the averaged amplitude of neural responses between the native and non-native groups, with the group having the higher amplitude value marked as dominant.

For the evaluation of backward models, permutation tests were applied to compare differences in reconstruction scores between the target and corresponding baseline models. A surrogate distribution of the differences was generated using 5,000 iterations. The significance of the difference was defined as the probability of the observed value under the surrogate distribution with the significance level of 0.05. Comparisons of the reconstruction scores between target models of native and non-native speakers were conducted using the same procedure.

## 3 Results

### 3.1 Differences in TRFs between native and non-native speakers

**Figure 2:**
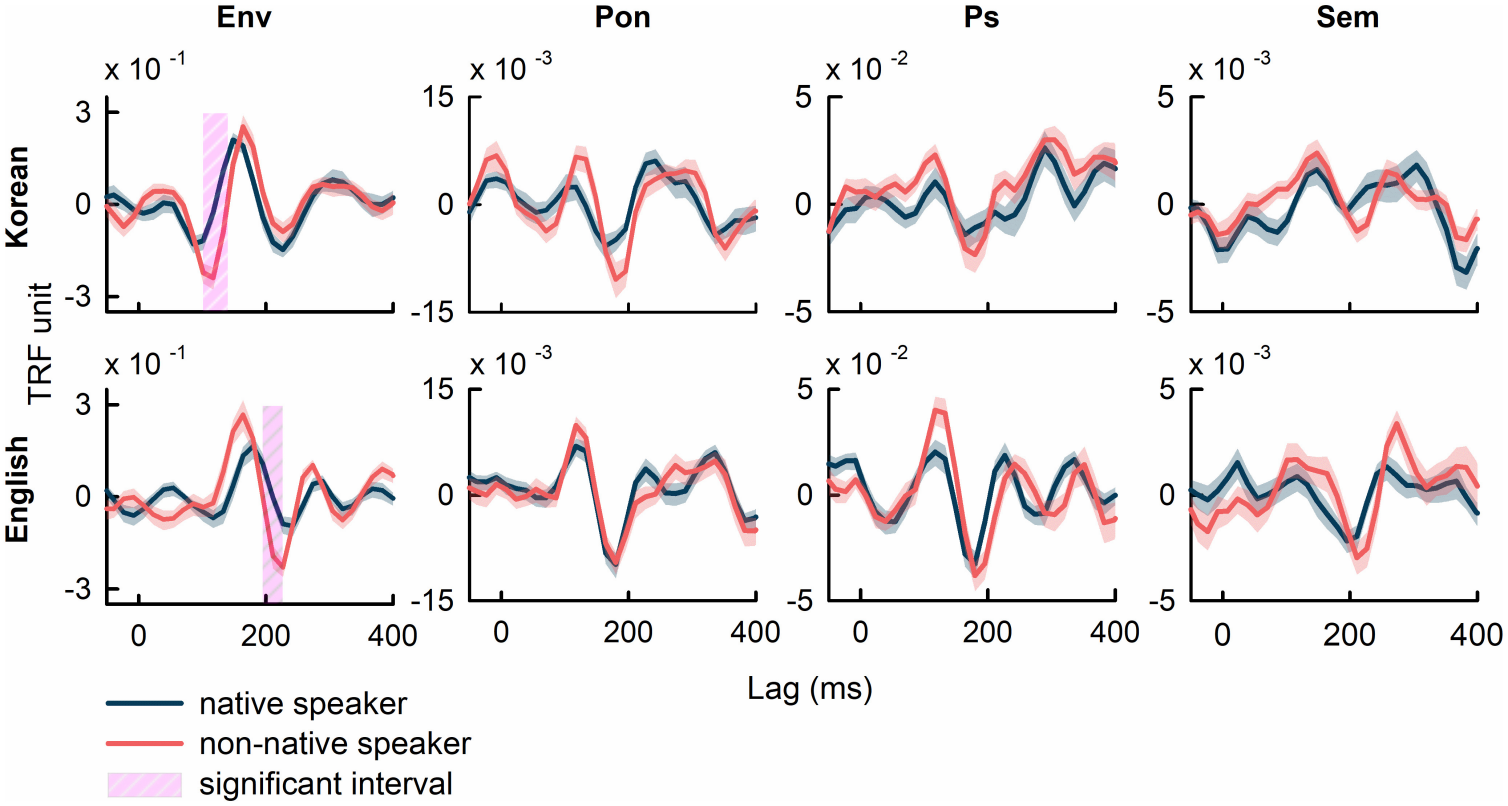
The grand average TRFs across all EEG channels and all participants computed using specific speech features of speech envelope (Env), phoneme onset (Pon), phonemic surprisal (Ps), and semantic dissimilarity (Sem). The intervals, which denotes significant difference between native and non-native speakers, were marked in pink shades (*p*<*0.01*, FDR-corrected). The shaded area in each plot represents the standard error of TRF weights.

Figure 2 illustrates the grand average TRFs, averaged across all electrodes and participants, for the speech features of speech envelope (Env), phoneme onset (Pon), phonemic surprisal (Ps), and semantic dissimilarity (Sem). Comparisons of TRF weights corresponding to the speech envelope between native and non-native speakers revealed significant differences (*p*<*0.01*, FDR-corrected). The interval showing significant differences in Korean occurs from 115 ms to 140 ms (17 out of 64 electrodes show significant differences; |t(38)| in the range of [3.63, 5.53], *p*<*0.01*, FDR-corrected), while in English, this interval spans from 200 ms to 220 ms (21/64 recording channels show differences; |t(38)| in the range of [3.46, 5.25], *p*<*0.01*, FDR-corrected). These intervals, defined as the longest consecutive time points where TRF weights exhibit a significant difference between native and non-native speakers, survived after FDR correction (see Supplementary Figure 1 and Supplementary Table 1 in the Supplementary Materials for additional details on TRF weights to the speech envelope in native speakers for Korean and English). No significant intervals were observed after FDR correction in comparisons between the TRF weights of native and non-native speakers corresponding to the other speech features.

**Figure 3:**
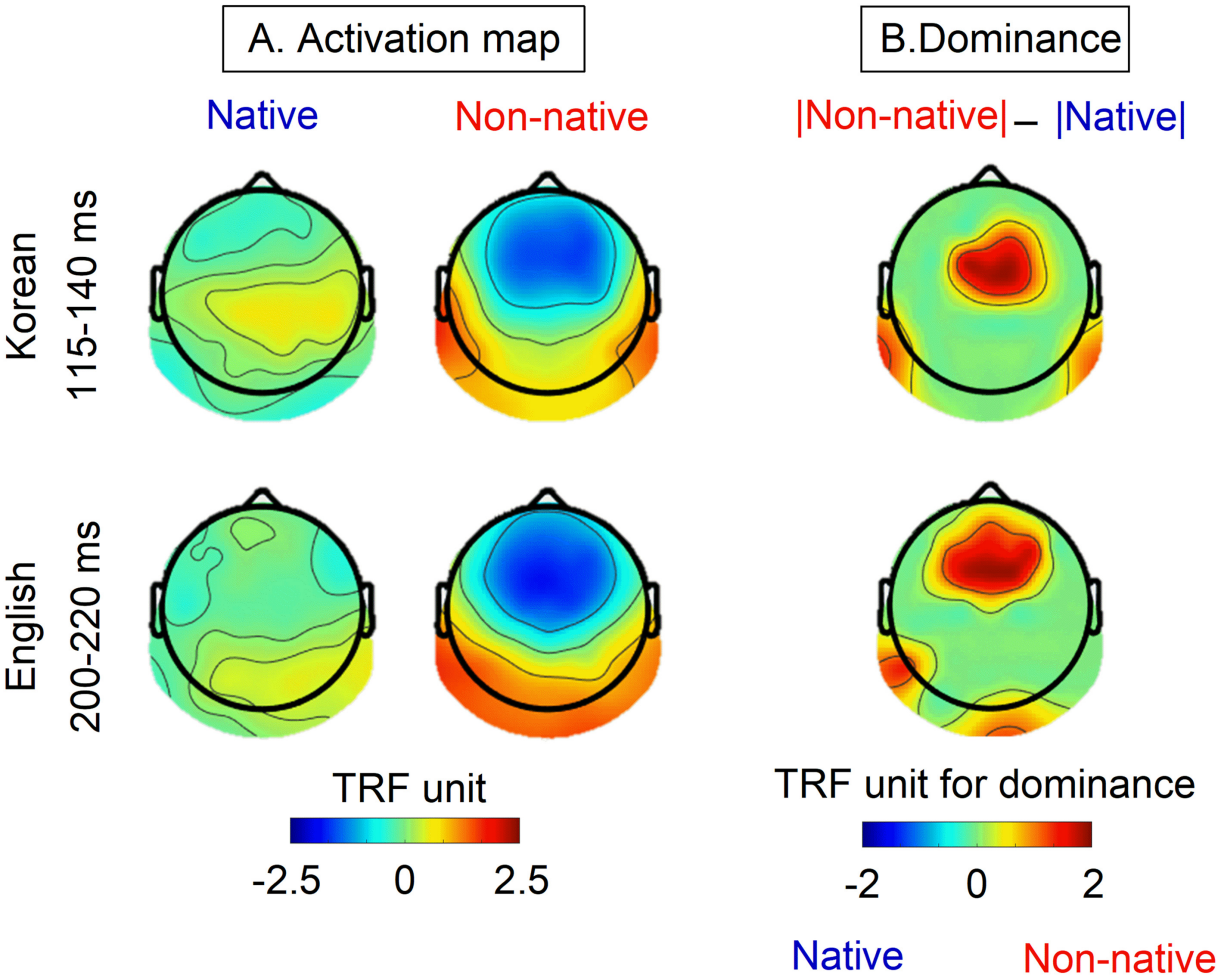
**(A)** The envelope-based TRFs activation map from native and non-native speakers and **(B)** the differences (dominacy) in TRF weights between non-native and native speakers. Activation maps were generated by averaging data within the specific intervals of 115-140 ms for Korean and 200-220 ms, corresponding to the shaded interval where significant differences were observed as illustrated in Figure 2.

Figure 3A represents the activation map of native and non-native speakers during the intervals that showed significant differences in TRF weights corresponding to the speech envelope. The dominance map highlights the differences in TRF weights between non-native and native speakers as seen in Figure 3B. For non-native speakers, TRF weights corresponding to the speech envelope show strong negative weights in the frontal-central area and strong positive weights in the bilateral temporal-parietal and occipital regions during both intervals. In contrast, moderated TRF weights were observed with a slightly positive response in the right temporal region and a negative response in the frontal area were observed in native speakers. The dominance map reveals stronger TRF weights for non-native speakers compared to those for native speakers. For non-native speakers, the dominant areas are consistently observed in the frontal, central, and temporal-parietal lobes in both the Korean and English cases.

**Figure 4:**
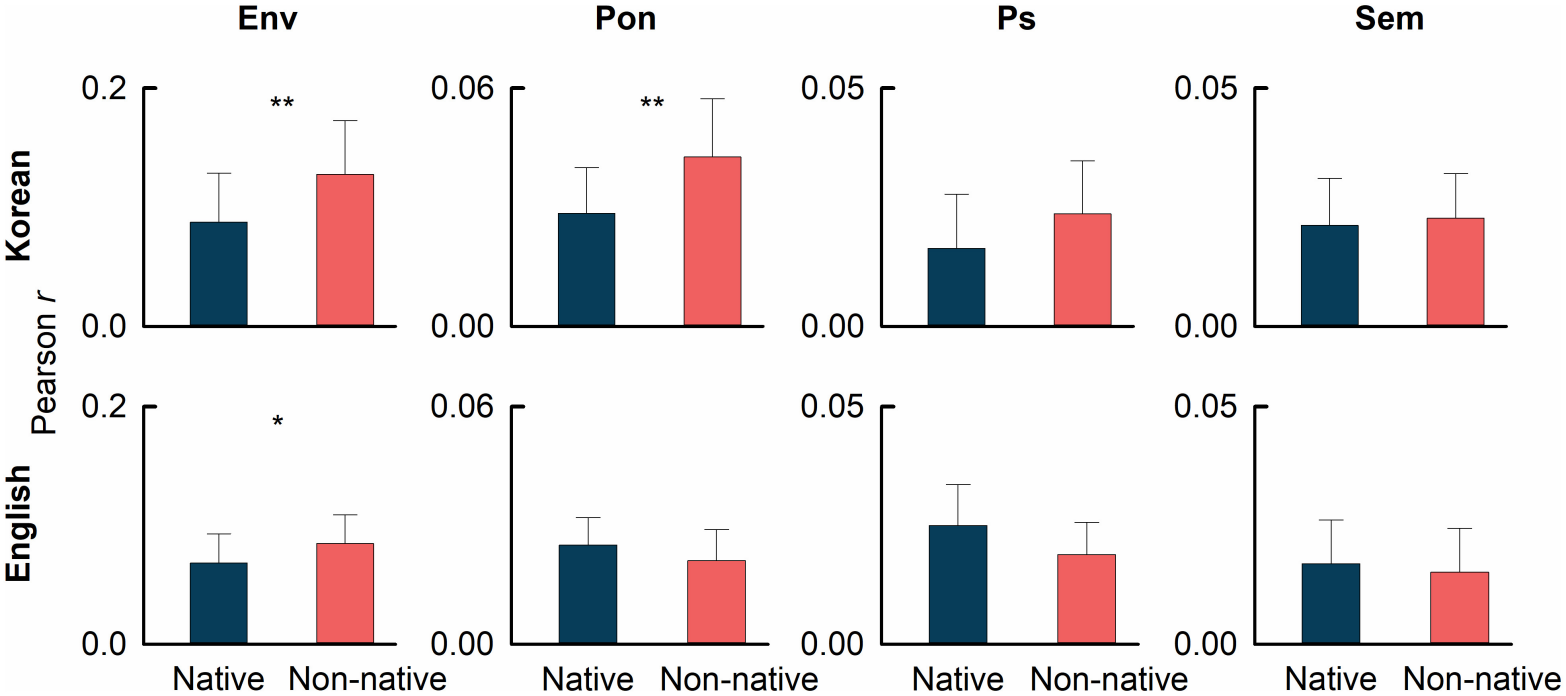
The reconstruction scores from backward models corresponding to the speech envelope (Env), phoneme onset (Pon), phonemic surprisal (Ps), and semantic dissimilarity (Sem) for Korean (upper panels) and English (lower panels) language. The significant difference in reconstruction scores between native and non-native speakers is denoted by *p < 0.05 and **p < 0.01.

Figure 4 provides the reconstruction scores computed from backward models. All target backward models corresponding to the speech features of speech envelope, phoneme onset, phonemic surprisal, and semantic dissimilarity are significantly robust to their corresponding baseline models (permutation test, *p*<*0.05*, data not shown). The reconstruction score for the speech envelope case was significantly higher for non-native speakers compared to that for native speakers for both Korean (permutation test, Hedges' *g* = 0.931, *p* = *0.002*) and English (permutation test, Hedges' *g* = 0.683, *p* = *0.022*) cases. The phoneme onset-based backward model showed a significant difference in reconstruction score between non-native and native speakers in the Korean case (permutation test, Hedges' *g* = 1.076, *p* = *0.001*), but not in the English case (permutation test, Hedges' *g* = 0.522, *p* = *0.944*). No significant differences in reconstruction scores for the speech features of phonemic surprisal and semantic dissimilarity were observed.

### 3.2 Differences in PRPs between native and non-native speakers

**Figure 5:**
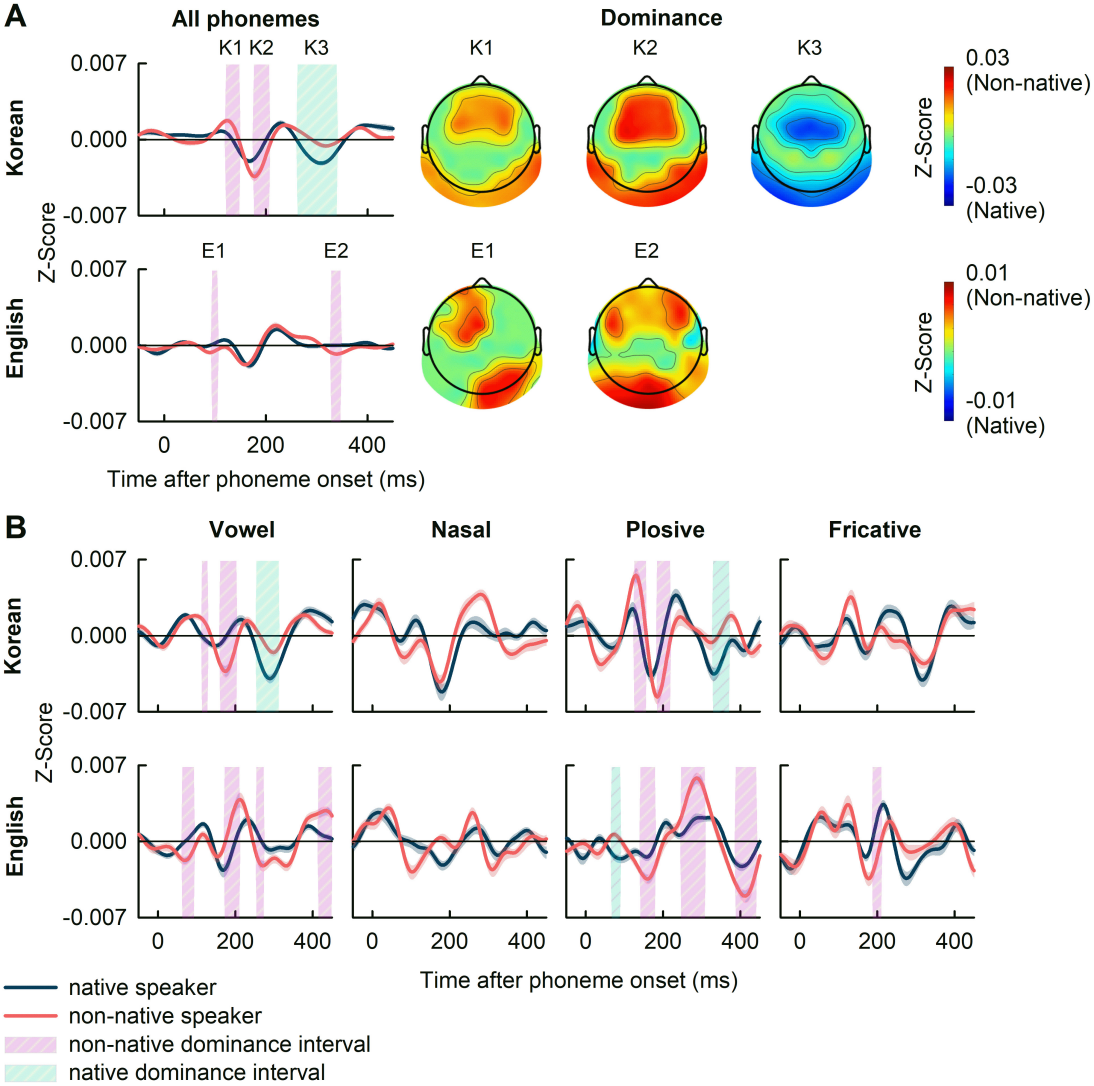
**(A)** The grand average of PRPs and the dominant topography in the intervals (K1, K2, and K3; E1 and E2) showing significant differences between native and non-native speakers. **(B)** The PRPs to specific phonemic categories of vowel, nasal, plosive, and fricative. The shaded areas denote intervals where significant differences between native and non-native speakers were observed (*p*<*0.01*, FDR-corrected). The shaded areas represent the standard error of PRPs.

Figure 5 presents the grand average PRPs across all phonemes (panel A), and the PRPs for all categories of speech sounds classified by vowel, nasal, plosive, and fricative (panel B). In Korean case, a comparison between the grand average PRPs of native and non-native speakers revealed significant differences, with non-native dominance during the intervals of 120–150 ms and 175–210 ms, and native dominance during the interval of 260–340 ms shaded and denoted as K1, K2, and K3, respectively (|t(38)| in the range of [3.32, 5.53], *p*<*0.01*, FDR-corrected). The frontal, frontal-central, temporal, and parietal areas of the brain showed non-native dominance, whereas the central, temporal, and parietal areas showed native dominance (Figure 5A). In English case, a comparison of the grand average PRPs across all phonemes showed significant non-native dominance in the intervals 90–100 ms and 330–350 ms, shaded and denoted as E1 and E2 areas, respectively (|t(38)| in the range of [3.18, 3.69], *p*<*0.01*, FDR-corrected). The significantly higher PRPs in non-native were observed in the left frontal, left frontal-central, and right temporal-parietal brain regions for E1 case, and the left and right frontal and left temporal-parietal regions for E2 case (Figure 5A).

In the comparison of PRPs according to phonemic categories, significant difference between native and non-native speakers were observed in various intervals as seen in Figure 5B. For Korean case, these differences occurred in the intervals: 115–130, 160–200, and 255–310 ms for vowels (|t(38)| in the range of [3.32, 5.98]); 120–150, 180–210, and 320–365 ms for plosives (|t(38)| in the range of [3.30, 4.80]); no significant intervals found for nasals and fricatives. In English case, significant intervals were observed at 65–90, 175–210, 255–270, and 420–445 ms for vowels (|t(38)| in the range of [3.32, 4.59]); 65–90, 140–180, 250–310, and 390–440 ms for plosives (|t(38)| in the range of [3.15, 5.13]); 190–210 ms for fricatives (|t(38)| in the range of [3.97, 4.40]); and none for nasals. Clearly, the range in which differences occurred varied depending on the phonemic category (see Supplementary Table 2 for a list of phonemes showing significant differences in the PRP analysis between native and non-native speakers).

## 4 Discussion

This study investigated neural response to speech in both English and Korean native speakers to cross-validate differences in speech perception across languages. Non-native speakers showed significantly enhanced neural tracking of the speech envelope compared to native speakers, whereas no group differences were observed for phoneme onset, phonemic surprisal, and semantic dissimilarity. These findings indicate that limited language experience in non-native speakers leads to enhanced neural tracking of bottom-up acoustic features, particularly the speech envelope, while higher level linguistic processing remains relatively inactive during passive listening. The PRP analyses resulted in significant group differences in the grand average PRPs across all phonemes, as well as within specific categories of vowels, plosives, and fricatives category. Overall, non-native speakers engaged stronger phonemic neural responses, likely due to exposure to unfamiliar phonemes in their native language.

These findings align with previous studies reporting enhanced neural tracking in non-native speakers (Reetzke et al., 2021; Song and Iverson, 2018; Zou et al., 2019). (Zou et al. 2019) demonstrated that individuals unfamiliar with the spoken speech demonstrate stronger neural tracking despite reduced performance in segment detecting tasks. (Reetzke et al. 2021) proposed that non-native speakers may engage attentional mechanisms to enhance sensitivity to low-level acoustic cues, regardless of speech contents. These suggest that enhanced envelope tracking among non-native speakers reflects a compensatory reliance on bottom-up acoustic cue, likely due to limited language experience. Moreover, although low-frequency neural responses have been known to track both acoustic and linguistic information in speech (i.e., reflecting the influence of top-down predictive processes on neural envelope tracking), the absence of significant differences in neural responses to phonemic surprisal and semantic dissimilarity further supports the hypothesis of a greater reliance on bottom-up acoustic cues in non-native speakers.

Regarding the discrepancy in the latency of significant periods observed in envelope tracking between the English and Korean cases (Figure 2), speech processing primarily occurs in common brain regions (e.g., Broca's and Wernicke's area). However, the comprehension of different languages relies on distinct interaction patterns among these regions (Ge et al., 2015; Li et al., 2021; Wei et al., 2023). The variation in these interactions may underlie differences in listening strategies, ultimately resulting in varying latencies of significant periods. This finding aligns with the current results, demonstrating a common dominance map (central and temporal areas) but distinct latencies between English and Korean (Figures 2, 3).

Analysis of TRF weights, focusing on the amplitude and latency of the N1, P2, and N2 peaks between the two language groups, reveals variations in speech comprehension across languages (Supplementary Figure 1). Notably, significant variations emerged in the latency of all three peaks (two-tailed unpaired *t*-test, *p*<*0.05*), whereas no significant difference was observed in the amplitude of the TRFs between English and Korean native speakers (Supplementary Table 1). The differences in the latency, especially in the N1 and P2 peaks, contribute to the evidence supporting the divergent listening strategies across different languages. These findings, coupled with the differences in the latency of significant periods in native vs. non-native analyses, collectively underscore the intricate and language-specific nature of the neural processes involved in speech comprehension.

At the phonemic level, though no significant difference was observed in TRF analysis for phoneme onset, PRPs analysis revealed significantly different intervals between native and non-native speakers in the grand average PRPs (Figure 5A). The differences were observed in brain regions distributed across the frontal, frontal-central, temporal, and parietal areas, with most of the significant intervals showing non-native dominance in both Korean and English. This discrepancy between TRF and PRP results can be attributed to methodological differences (Khalighinejad et al., 2017). Specifically, ridge regularization in TRF estimation can introduce spurious TRF responses (Kulasingham and Simon, 2022) whereas the time-locked averaging approach in PRP analysis preserves the precise temporal features of neural responses. The phonemes closely resembled the Korean phonemes that are absent in English and vice versa likely to induce significant differences in neural response between native and non-native speakers (Supplementary Table 2; Cho and Park, 2006). For instance, the consonant /B/ is present in English but not in Korean, or English phonemic vowels exhibit finer-grained distinctions than their Korean counterparts, as seen in the pair /UW/ and /UH/, where /UW/ represents a tense version of /UH/. These results may explain the significant differences in PRPs observed in response to different phoneme groups (Figure 5B), suggesting that non-native speakers may engage more with phonemes not present in their first language, resulting in stronger PRP responses to phoneme classes with more unfamiliar phonemes.

However, while the patterns and latencies of significantly different intervals in the Korean case are consistent across the grand average PRPs and the PRPs in response to vowels and plosives, these attributes vary considerably in the English case, particularly across the grand average PRPs and the PRPs in response to vowels, plosives, and fricatives. This variation may be due to differences in experience levels of non-native speakers in Korean and English. This gap can cause a difference in the analysis because non-native speakers with higher experience tend to exhibit neural tracking more similar to that of native speakers (Di Liberto et al., 2021). This discrepancy may arise from the difference in experience in non-native language such that, compared to English native speakers, Korean native speakers generally encounter higher opportunities to engage with English due to education and sociocultural environment. This disparity in language experience was reflected in the similarity between the grand average PRPs from native and non-native speakers, which was lower for Korean (*r* = 0.636) than for English (*r* = 0.799). Nevertheless, the effect of language experience on PRPs in non-native speakers still remains insufficiently understood and warrants further systematical exploration. This observation also raises questions about the impact of language-specific phonemic characteristics on temporal processing dynamics, emphasizing the role of phoneme class in shaping response patterns among native and non-native speakers.

While this study revealed clear differences in neural tracking of the speech envelope and phoneme processing between native and non-native speakers, the absence of formal measures of language proficiency warrants consideration. Since non-native speakers with varying proficiency levels may employ different strategies to process speech stimuli, future research should include explicit measures of language proficiency to better examine its influence on the processing of bottom-up acoustic features of speech.

In summary, this study demonstrated that non-native speakers with lack of language experience rely more on acoustic cues during speech comprehension, as evidenced by significantly enhanced neural tracking of the speech envelope. This is consistent with the incomplete language model suggesting that non-native speakers prioritize the encoding of acoustic features (Mattys et al., 2010, 2013). The results align with the growing consensus that while directing attention to speech enhances neural tracking of the envelope, this enhancement does not necessarily translate to better comprehension. For non-native speakers, increased envelope tracking likely reflects a reliance on low-level auditory features in the absence of robust top-down linguistic support. These bottom-up strategies may differ depending on the listener's native language. In addition, distinct phonemic-level processing patterns observed in the PRP analyses further underscore the role of language experience in shaping auditory perception. Finally, this study demonstrates the feasibility of combining PRP and TRF analyses to investigate complementary neural mechanisms during continuous speech perception, with TRFs capturing neural tracking of speech features and PRPs revealing phoneme-specific neural dynamics.

## Data Availability

The data used to support the findings of this study are available from the corresponding author upon request.

## References

[B1] Abrams D. A. Nicol T. Zecker S. Kraus N. (2008). Right-hemisphere auditory cortex is dominant for coding syllable patterns in speech. J. Neurosci. 28, 3958–3965. 10.1523/JNEUROSCI.0187-08.2008 18400895 PMC2713056

[B2] Ahissar E. Nagarajan S. Ahissar M. Protopapas A. Mahncke H. Merzenich M. M. (2001). Speech comprehension is correlated with temporal response patterns recorded from auditory cortex. Proc. Nat. Acad. Sci. 98, 13367–13372. 10.1073/pnas.201400998 11698688 PMC60877

[B3] Aiken S. J. Picton T. W. (2008). Human cortical responses to the speech envelope. Ear Hear. 29, 139–157. 10.1097/AUD.0b013e31816453dc 18595182

[B4] Aldag N. Nogueira W. (2024). Phoneme-related potentials recorded from normal hearing listeners and cochlear implant users in a selective attention paradigm to continuous speech. Hear. Res. 454:109136. 10.1016/j.heares.2024.109136 39532054

[B5] Babcock L. Stowe J. C. Maloof C. J. Brovetto C. Ullman M. T. (2012). The storage and composition of inflected forms in adult-learned second language: a study of the influence of length of residence, age of arrival, sex, and other factors. Biling. Lang. Cogn. 15, 820–840. 10.1017/S1366728912000053

[B6] Benjamini Y. Hochberg Y. (1995). Controlling the false discovery rate: a practical and powerful approach to multiple testing. J. Royal Stat. Soc. Series B 57, 289–300. 10.1111/j.2517-6161.1995.tb02031.x

[B7] Benjamini Y. Yekutieli D. (2001). The control of the false discovery rate in multiple testing under dependency. Ann. Stat. 29, 1165–1188. 10.1214/aos/101369999838281721

[B8] Bilger R. C. Nuetzel J. M. Rabinowitz W. M. Rzeczkowski C. (1984). Standardization of a test of speech perception in noise. J. Speech Lang. Hear. Res. 27, 32–48. 10.1044/jshr.2701.32 6717005

[B9] Boersma P. (2001). Praat, a system for doing phonetics by computer. Glot Int. 5, 341–345.31350110

[B10] Borghini G. Hazan V. (2018). Listening effort during sentence processing is increased for non-native listeners: a pupillometry study. Front. Neurosci. 12:152. 10.3389/fnins.2018.00152 29593489 PMC5859302

[B11] Brandmeyer A. Farquhar J. D. McQueen J. M. Desain P. W. J. (2013). Decoding speech perception by native and non-native speakers using single-trial electrophysiological data. PLoS ONE 8:e68261. 10.1371/journal.pone.0068261 23874567 PMC3708957

[B12] Broderick M. P. Anderson A. J. Di Liberto G. M. Crosse M. J. Lalor E. C. (2018). Electrophysiological correlates of semantic dissimilarity reflect the comprehension of natural, narrative speech. Curr. Biol. 28, 803–809. 10.1016/j.cub.2018.01.080 29478856

[B13] Browne M. W. (2000). Cross-validation methods. J. Math. Psychol. 44, 108–132. 10.1006/jmps.1999.1279 10733860

[B14] Brysbaert M. New B. (2009). Moving beyond Kučera and Francis: a critical evaluation of current word frequency norms and the introduction of a new and improved word frequency measure for American English. Behav. Res. Methods 41, 977–990. 10.3758/BRM.41.4.977 19897807

[B15] Cho J. Park H.-K. (2006). A comparative analysis of Korean-English phonological structures and processes for pronunciation pedagogy in interpretation training. Meta 51, 229–246. 10.7202/013253ar

[B16] Coughlin C. E. Fiorentino R. Royle P. Steinhauer K. (2019). Sensitivity to inflectional morphology in a non-native language: Evidence from ERPs. Front. Commun. (Lausanne) 4:21. 10.3389/fcomm.2019.00021

[B17] Crosse M. J. Di Liberto G. M. Bednar A. Lalor E. C. (2016). The multivariate temporal response function (mTRF) toolbox: a A MATLAB toolbox for relating neural signals to continuous stimuli. Front. Hum. Neurosci. 10:604. 10.3389/fnhum.2016.00604 27965557 PMC5127806

[B18] Das N. Vanthornhout J. Francart T. Bertrand A. (2020). Stimulus-aware spatial filtering for single-trial neural response and temporal response function estimation in high-density EEG with applications in auditory research. NeuroImage 204:116211. 10.1016/j.neuroimage.2019.116211 31546052 PMC7355237

[B19] Delorme A. Makeig S. (2004). EEGLAB: an open source toolbox for analysis of single-trial EEG dynamics including independent component analysis. J. Neurosci. Methods 134, 9–21. 10.1016/j.jneumeth.2003.10.009 15102499

[B20] Di Liberto G. M. Nie J. P. Yeaton J. Khalighinejad B. Shamma S. A. Mesgarani N. (2021). Neural representation of linguistic feature hierarchy reflects second-language proficiency. NeuroImage 227:117616. 10.1016/j.neuroimage.2020.117586 33346131 PMC8527895

[B21] Di Liberto G. M. O'Sullivan J. A. Lalor E. C. (2015). Low-frequency cortical entrainment to speech reflects phoneme-level processing. Curr. Biol. 25, 2457–2465. 10.1016/j.cub.2015.08.030 26412129

[B22] Ding N. Melloni L. Zhang H. Tian X. Poeppel D. (2016). Cortical tracking of hierarchical linguistic structures in connected speech. Nat. Neurosci. 19, 158–164. 10.1038/nn.4186 26642090 PMC4809195

[B23] Ding N. Simon J. Z. (2014). Cortical entrainment to continuous speech: functional roles and interpretations. Front. Hum. Neurosci. 8:311. 10.3389/fnhum.2014.00311 24904354 PMC4036061

[B24] Etard O. Ben Messaoud R. Gaugain G. Reichenbach T. (2022). No evidence of attentional modulation of the neural response to the temporal fine structure of continuous musical pieces. J. Cogn. Neurosci. 34, 411–424. 10.1162/jocn_a_01811 35015867

[B25] Fuhrmeister P. Phillips M. C. McCoach D. B. Myers E. B. (2023). Relationships between native and non-native speech perception. J. Exp. Psychol. Learn. Memory Cogn. 49, 1161–1179. 10.1037/xlm0001213 36757985

[B26] Ge J. Q. Peng G. Lyu B. Wang y. Zhuo Y. Niu Z.. (2015). Cross-language differences in the brain network subserving intelligible speech. Proc. Natl. Acad. Sci. USA. 112, 2972–2977. 10.1073/pnas.1416000112 25713366 PMC4364212

[B27] Grave E. Bojanowski P. Gupta P. Joulin A. Mikolov T. (2018). “Learning word vectors for 157 languages,” in Proceedings of the Eleventh International Conference on Language Resources and Evaluation (LREC 2018). (Paris: European Language Resources Association), 3483–3487.

[B28] Ihara A. S. Matsumoto A. Ojima S. Katayama J. Nakamura K.. (2021). Prediction of second language proficiency based on electroencephalographic signals measured while listening to natural speech. Front. Hum. Neurosci. 15:665809. 10.3389/fnhum.2021.665809 34335208 PMC8322447

[B29] Jang H. and Lee, J-B.. (2008). Development of Korean standard sentence lists for sentence recognition tests. Audiology 4, 161–177. 10.21848/audiol.2008.4.2.161

[B30] Jeon M. J. Woo J. (2023). Effect of speech-stimulus degradation on phoneme-related potential. PLoS ONE 18:e0285990. 10.1371/journal.pone.0287584 37352220 PMC10289326

[B31] Keitel A. Gross J. Kayser C. (2018). Perceptually relevant speech tracking in auditory and motor cortex reflects distinct linguistic features. PLoS Biol. 16:e2004473. 10.1371/journal.pbio.2004473 29529019 PMC5864086

[B32] Khalighinejad B. da Silva G. C. Mesgarani N. (2017). Dynamic encoding of acoustic features in neural responses to continuous speech. J. Neurosci. 37, 2176–2185. 10.1523/JNEUROSCI.2383-16.2017 28119400 PMC5338759

[B33] Kisler T. Reichel U. Schiel F. (2017). Multilingual processing of speech via web services. Comput. Speech Lang. 45, 326–347. 10.1016/j.csl.2017.01.005

[B34] Kulasingham J. P. Simon J. Z. (2022). Algorithms for estimating time-locked neural response components in cortical processing of continuous speech. IEEE Transact. Biomed. Eng. 70, 88–96. 10.1101/2022.01.18.476815 35727788 PMC9946293

[B35] Li Y. Tang C. Lu J. Wu J. Chang E. F. (2021). Human cortical encoding of pitch in tonal and non-tonal languages. Nat. Commun. 12:1161. 10.1038/s41467-021-21430-x 33608548 PMC7896081

[B36] Marmarelis V. Z. (2004). Nonlinear Dynamic Modeling of Physiological Systems. (Hoboken, NJ: Wiley) 10.1002/9780471679370

[B37] Martin B. A. Tremblay K. L. Korczak P. (2008). Speech evoked potentials: from the laboratory to the clinic. Ear Hear. 29, 285–313. 10.1097/AUD.0b013e3181662c0e 18453883

[B38] Mattys S. L. Carroll L. M. Li C. K. W. Chan S. L. Y. (2010). Effects of energetic and informational masking on speech segmentation by native and non-native speakers. Speech Commun. 52, 887–899. 10.1016/j.specom.2010.01.005

[B39] Mattys S. L. Davis M. H. Bradlow A. R. Scott S. K. (2013). “Speech recognition in adverse conditions: a review,” in Speech Recognition in Adverse Conditions: Explorations in Behaviour and Neuroscience, eds. S. L. Mattys, A. R. Bradlow, M. H. Davis, and S. K. Scott (Psychology Press), 1–26. 10.4324/9781315825083

[B40] Mesgarani N. Chang E. F. (2012). Selective cortical representation of attended speaker in multi-talker speech perception. Nature 485, 233–236. 10.1038/nature11020 22522927 PMC3870007

[B41] Millman R. E. Prendergast G. Hymers M. Green G. G. (2012). Representations of the temporal envelope of sounds in human auditory cortex: can the results from invasive intracortical “depth” electrode recordings be replicated using non-invasive MEG “virtual electrodes”? Neuroimage 64, 185–196. 10.1016/j.neuroimage.2012.09.017 22989625

[B42] Mueller J. L. Hirotani M. Friederici A. D. (2007). ERP evidence for different strategies in the processing of case markers in native speakers and non-native learners. BMC Neurosci. 8:56. 10.1186/1471-2202-8-18 17331265 PMC1828061

[B43] Muncke J. Kuruvila I. Hoppe U. (2022). Prediction of speech intelligibility by means of EEG responses to sentences in noise. Front. Neurosci. 16:876421. 10.3389/fnins.2022.876421 35720724 PMC9198593

[B44] Muralimanohar R. K. Kates J. M. Arehart K. H. (2017). Using envelope modulation to explain speech intelligibility in the presence of a single reflection. J. Acoust. Soc. Am. 141, EL482–EL487. 10.1121/1.4983630 28599537

[B45] Obleser J. Kayser C. (2019). Neural entrainment and attentional selection in the listening brain. Trends Cogn. Sci. 23, 913–926. 10.1016/j.tics.2019.08.004 31606386

[B46] O'Sullivan J. A. Power A. J. Mesgarani N. Rajaram S. Foxe J. J. Shinn-Cunningham B. G.. (2015). Attentional selection in a cocktail party environment can be decoded from single-trial EEG. Cereb. Cortex 25, 1697–1706. 10.1093/cercor/bht355 24429136 PMC4481604

[B47] Reetzke R. Gnanateja G. N. Chandrasekaran B. (2021). Neural tracking of the speech envelope is differentially modulated by attention and language experience. Brain Lang. 213:104891. 10.1016/j.bandl.2020.104891 33290877 PMC7856208

[B48] Ringach D. Shapley R. (2004). Reverse correlation in neurophysiology. Cogn. Sci. 28, 147–166. 10.1207/s15516709cog2802_2

[B49] Slaats S. Weissbart H. Schoffelen J. M. Meyer A. S. Martin A. E. (2023). Delta-band neural responses to individual words are modulated by sentence processing. J. Neurosci. 43, 4867–4883. 10.1523/JNEUROSCI.0964-22.2023 37221093 PMC10312058

[B50] Song J. Iverson P. (2018). Listening effort during speech perception enhances auditory and lexical processing for non-native listeners and accents. Cognition 179, 163–170. 10.1016/j.cognition.2018.06.001 29957515

[B51] Sun L. Li C. Wang S. Si Q. Lin M. Wang N.. (2023). Left frontal eye field encodes sound locations during passive listening. Cereb. Cortex 33, 3067–3079. 10.1093/cercor/bhac261 35858212

[B52] Tang K. de Chene B. (2014). “A new corpus of colloquial Korean and its applications,” in The 14th Conference on Laboratory Phonology. (Tokyo: LabPhon).

[B53] Vanthornhout J. Decruy L. Francart T. (2019). Effect of task and attention on neural tracking of speech. Front. Neurosci. 13:977. 10.3389/fnins.2019.00977 31607841 PMC6756133

[B54] Wei X. Adamson H. Schwendemann M. Goucha T. Anwander A.. (2023). Native language differences in the structural connectome of the human brain. NeuroImage 270:119955. 10.1016/j.neuroimage.2023.119955 36805092

[B55] Winkler I. Kujala T. Tiitinen H. Sivonen P. Czigler I.. (1999). Brain responses reveal the learning of foreign language phonemes. Psychophysiology 36, 638–642. 10.1111/1469-8986.3650638 10442032

[B56] Yoon T.-J. Kang Y. (2014). Monophthong analysis on a large-scale speech corpus of read-style Korean. Phonet. Speech Sci. 6, 139–145. 10.13064/KSSS.2014.6.3.139

[B57] Zou J. J. Feng J. Xu T. Jin P. Luo C.. (2019). Auditory and language contributions to neural encoding of speech features in noisy environments. NeuroImage 192, 66–75. 10.1016/j.neuroimage.2019.02.047 30822469

