# Supplementary material for: Differential electroencephalography responses in speech perception between native and non-native speakers
Source: Front Hum Neurosci. 2025 Oct 24;19:1661010. doi: 10.3389/fnhum.2025.1661010 (PMC12592156; doi:10.3389/fnhum.2025.1661010)
Supplement: Supplementary file 1 [file Data_Sheet_1.docx]

Supplementary Material

# Supplementary Figures and Tables

## Supplementary Figures

**Figure S1.** The grand average TRFs corresponding to the speech envelope, averaged across all electrodes, of native speakers in both Korean and English. The significant difference in the peak latency between Korean native speakers and English native speakers is denoted by * (*p<0.05*) and ** (*p<0.01*). The shaded areas represent the standard error of TRF weights.





## Supplementary Tables

**Table S1.** The mean amplitude and latency of the N1, P2, and N2 peaks of the TRFs corresponding to the speech envelope across all participants and the corresponding p‐value from a two‐tailed unpaired *t*‐test between Korean and English native speakers

|  | N1 | | P2 | | N2 | |
| --- | --- | --- | --- | --- | --- | --- |
|  | Mean amplitude (TRF unit) | Mean latency (ms) | Mean amplitude (TRF unit) | Mean latency (ms) | Mean amplitude (TRF unit) | Mean latency (ms) |
| Korean native speakers | −0.174 | 98.9 | 0.193 | 159.4 | −0.177 | 214.3 |
| English native speakers | −0.167 | 122.3 | 0.199 | 178.1 | −0.14 | 229.3 |
| *t‐*value | 0.01 | 3.15 | 0.80 | 3.04 | 1.13 | 2.13 |
| *p*‐value | 0.990 | 0.003 ** | 0.430 | 0.004 ** | 0.264 | 0.039 * |

Significant difference level was denoted by * (*p*<0.05) and ** (*p<0.0*1).

**Table S2.** List of phonemes showing significant differences in PRP analysis between native and non‐native speakers

| Phoneme | | | | | | | |
| --- | --- | --- | --- | --- | --- | --- | --- |
| Korean sentences | | | | English sentences | | | |
| Vowel | Nasal | Plosive | Fricative | Vowel | Nasal | Plosive | Fricative |
| A | L^+^ | P^+^ | J | AH^+^ | R^+^ | B^+^ | CH^+^ |
| E |  | KK^+^ |  | IH |  | T | HH |
| I |  |  |  | UH^+^ |  | D^+^ | F^+^ |

^+^ denotes that phoneme is available in only the Korean or English language (Cho & Park, 2006).
